# SERVICE AND PROGRAM NEEDS TO SUPPORT OLDER, LOW-INCOME HOMEOWNERS AGING IN PLACE

**DOI:** 10.1093/geroni/igae098.3891

**Published:** 2024-12-31

**Authors:** Sung-Jin Lee, Renee Robinson, Elizabeth Hopfer, Minyong Lee, Kathleen Parrott

**Affiliations:** North Carolina A&T State University, Greensboro, North Carolina, United States; North Carolina A&T State University, Greensboro, North Carolina, United States; North Carolina A&T State University, Greensboro, North Carolina, United States; North Carolina A&T State University, Greensboro, North Carolina, United States; Virginia Tech, Blacksburg, Virginia, United States

## Abstract

An interdisciplinary team of the Housing, Kinesiology, and Textile programs at North Carolina A&T State University is collaborating on a multi-year research project to examine the person-environment fit perspective for older, low-income homeowners in North Carolina. Using a qualitative phenomenological study approach, researchers conducted 19 in-home and personal interviews with urban low-income, older homeowners in community-dwelling units. A local nonprofit housing agency providing home modification services to low-income older adults assisted with sample recruitment. Researchers conducted a content analysis using interview transcripts from recorded voices, home assessment data, photos taken during home visits, and interviewers’ notes. Data analysis revealed personal elements (e.g., living alone, informal support, mortgage payments, technology capability levels) and environmental elements (e.g., steps, dust/unclean home conditions, clutter, continuous upkeep needs) that can be associated with respondents’ aging-in-place practices. The qualitative data also highlighted the need for additional services and programs to improve their home environment or housing conditions beyond home modifications. Examples include cleaning services (e.g., dust on floors, carpets, ceilings, or vents), laundry services for home textiles (e.g., fabric drapes on windows), lawn care programs, and services for placing trash bins at the curb for pickup and returning them near the home. This study provides an opportunity to understand older, low-income homeowners aging in place and their home environments, which have been less explored using a qualitative approach. This research presentation will be supported by qualitative comments from study participants, along with photos of home environments.

